# Influence of plasticizer type on the structure and drug release characteristics of LM-pectin hydrogels

**DOI:** 10.55730/1300-0527.3775

**Published:** 2025-11-25

**Authors:** Banu KOCAAĞA, Fatma Seniha GÜNER

**Affiliations:** 1 Department of Chemical Engineering, Faculty of Chemical and Metallurgical Engineering, İstanbul Technical University, İstanbul, Turkiye; 2 Sabancı University Nanotechnology Research and Application Center (SUNUM), Sabancı University, İstanbul, Turkiye

**Keywords:** LM-pectin, plasticizer, hydrogel, controlled release, drug release kinetics

## Abstract

This study investigated the effects of different plasticizers—castor oil (CO), polyvinylpyrrolidone (PVP), and polyethylene glycol (PEG) with varying molecular weights (MWs)—on the structure and drug-release performance of low-methoxyl pectin (LM-pectin) hydrogels. Theophylline was used as a model drug to evaluate release behavior under physiologically relevant conditions. The incorporation of plasticizers modulated polymer–polymer interactions, swelling behavior, and thermal properties, thereby affecting drug-release kinetics. CO, a hydrophobic triglyceride, created microdomain-induced diffusion pathways; PVP, containing water-affinitive lactam units, facilitated moisture-driven plasticization; and PEG (MW 400/1000/1500), with hydroxyl-terminated chains, established hydrogen bonds with pectin. Structural analyses (FTIR and DSC) revealed that CO disrupted pectin packing, leading to a flexible yet crystalline matrix and enabling the highest cumulative drug release. PVP-based hydrogels exhibited enhanced crystallinity and slower release, whereas PEG formulations showed molecular-weight-dependent behavior. Kinetic calculations confirmed similar patterns, demonstrating non-Fickian transport for PEG400 and PEG1000 (diffusion associated with polymer relaxation) and an additional diffusion-limited profile for PEG1500 attributed to network densification. Among the methods evaluated, CO improved cumulative release, while PEG1500 and PVP promoted extended, lower-rate delivery. The selection of plasticizers must correspond with the design objective: CO for high cumulative release and PEG1500 or PVP for prolonged, diffusion-controlled administration. These results highlight the critical role of the plasticizer type in tailoring hydrogel performance. The LM-pectin formulations developed herein demonstrate potential for application in controlled dermal and mucosal drug-delivery systems.

## 1 Introduction

Hydrogels have attracted significant interest in the pharmaceutical and biomedical fields owing to their capacity to encapsulate and modulate the release of therapeutic agents. The controlled release behavior of hydrogels is governed by various factors, including polymer composition [1], cross-link density [2], porosity [3], crystallinity [4], and the inclusion of plasticizers [5,6]. Among naturally derived polymers, pectin is particularly notable for its excellent biocompatibility, biodegradability, and nontoxic nature, making it a highly promising candidate for biomedical and pharmaceutical applications. Low-methoxy pectin (LM-pectin), characterized by a degree of esterification below 50%, forms a stable three-dimensional network upon interaction with divalent cations such as Ca^2+^. This ionic crosslinking results in the formation of an “egg-box” structure, in which Ca^2+^ ions coordinate with the free carboxyl groups of adjacent pectin chains to generate cooperative junction zones [1]. These junction zones reinforce the hydrogel matrix by enhancing mechanical stability and reducing solubility, while simultaneously producing aqueous channels that regulate hydration, polymer mobility, and drug diffusion [2,3]. However, LM-pectin hydrogels frequently suffer from inherent brittleness, which is primarily attributed to the dense network of hydrogen bonds and strong cooperative interactions between polymer chains that restrict chain mobility and deformability [4]. These strong interactions limit chain mobility, resulting in stiff, fracture-prone films [5,6]. Therefore, overcoming brittleness is essential for enhancing the mechanical robustness of LM-pectin hydrogels and expanding their applicability in advanced biomedical fields, including wound healing, tissue engineering, and controlled drug delivery systems.

A well-established strategy to overcome brittleness in polysaccharide-based hydrogels is the incorporation of plasticizers, which weaken polymer–polymer interactions while forming new polymer–plasticizer linkages, thereby enhancing chain mobility [7]. By disrupting intermolecular hydrogen bonding, plasticizers improve the flexibility, ductility, and extensibility of hydrogel films [8]. This modulation of the polymer network strengthens mechanical properties and enhances the overall functionality of hydrogels, making them more adaptable for advanced biomedical applications [9,10].

Achieving the desired flexibility and release kinetics in hydrogels requires a delicate balance between polymer–polymer, polymer–plasticizer, and plasticizer–plasticizer interactions [11,12]. Beyond mechanical enhancements, plasticizers significantly influence porosity, diffusional pathways, and swelling behavior, all of which directly modulate drug release profiles [13–16]. Optimizing these parameters is critical when developing hydrogels for advanced biomedical applications, including controlled drug delivery systems and tissue engineering scaffolds.

Water is often regarded as the simplest and most ubiquitous plasticizer for hydrophilic polymer systems. Its exceptionally low glass transition temperature (approximately –135 °C) enables it to markedly soften polymer matrices by increasing chain mobility [17]. However, water can also form extensive hydrogen-bonding networks, which, under certain conditions, may reinforce existing polymer–polymer interactions rather than disrupt them—resulting in antiplasticizing effects [18]. Beyond water, various polar and nonpolar compounds function as plasticizers, each exerting distinct influences on mechanical strength and drug release. Polar plasticizers, such as polyols (glycerol and sorbitol) and carboxylic acids, can strengthen films by forming robust hydrogen bonds with polymer chains, while nonpolar plasticizers, such as certain oils, can enhance matrix flexibility [1,19,20]. The overall plasticizing efficacy depends on both the chemical structure of the plasticizer and its compatibility with the polymer [8,21]. Incompatible plasticizers tend to phase-separate during film formation, often resulting in poor mechanical integrity and compromised performance.

Castor oil (CO) has gained attention as a renewable, hydrophobic plasticizer due to its versatile chemical structure, which includes ester and hydroxyl groups as well as carbon–carbon double bonds, enabling various modifications and applications [22,23]. Extracted from *Ricinus communis*, its hydrophobic nature effectively fills microvoids within polymer matrices, reducing water retention and extending drug release durations [24,25]. Chen et al. [26] synthesized a CO-based diglycidyl ester plasticizer from ricinoleic acid, demonstrating its ability to improve the compatibility and flexibility of polyvinyl chloride matrices. Chakraborty et al. [27] demonstrated that CO can increase rigidity in specific polymeric systems via hydrophobic interactions, highlighting its dual functionality as both a plasticizer and structural modifier. These findings demonstrate the advantages of using CO for fine-tuning the mechanical performance and drug release kinetics of polymer-based delivery platforms. Compared to other vegetable oil-derived plasticizers, such as linseed or soybean oil, CO offers enhanced versatility due to its unique chemical structure, particularly the presence of hydroxyl and ester functionalities, which enable both hydrogen bonding and hydrophobic domain formation within the polymer matrix.

Polyethylene glycol (PEG), also widely used, is a particularly effective hydrophilic plasticizer for polysaccharide-based hydrogels [28,29]. The PEG chain length, or molecular weight (MW), determines its plasticizing efficiency, with lower MW generally providing greater chain mobility and higher MW contributing to improved structural stability [30]. The hydroxyl end groups of PEG can readily form hydrogen bonds with the polymer, disrupting rigid hydrogen-bonding regions and imparting elasticity to the film [29,31,32].

Polyvinylpyrrolidone (PVP) is a synthetic, water-soluble polymer consisting of repeating N-vinylpyrrolidone units. While not conventionally classified as a plasticizer, its lactam ring exhibits a strong affinity for water molecules, facilitating moisture absorption within hydrophilic polymer matrices [33,34]. This water-mediated plasticization mechanism enhances chain mobility, imparting increased flexibility and absorbent properties to the film, thereby improving its mechanical performance and processability [20].

The present study investigated the effects of four distinct plasticizers, namely CO, PEG of three MWs (400, 1000, and 1500 g/mol), and PVP, on the physicochemical properties and drug release behavior of LM-pectin hydrogel films. These plasticizers differ in polarity, functional groups, and molecular size, each contributing uniquely to modifications in film flexibility, permeability, and release dynamics. Theophylline, a widely used therapeutic agent for respiratory disorders, is utilized as a model drug to assess release kinetics and the extent of controlled delivery [1]. By investigating the interplay between plasticizer type, structural modifications, and drug diffusion mechanisms, the aim in the present study was to inform the rational design of LM-pectin-based hydrogels for advanced pharmaceutical and biomedical applications.

## 2 Materials and methods

### 2.1. Materials

LM-amide pectin was provided by Herbstreith & Fox KG (Germany). Tris (2-amino-2-(hydroxymethyl)propane-1,3-diol) and calcium chloride dihydrate (CaCl_2_·2H_2_O), along with theophylline, were purchased from Sigma-Aldrich (Düsseldorf, Germany). All chemicals used were of analytical grade and were utilized without further purification. Throughout the experiments, double-deionized water was used as the solvent.

### 2.2. Preparation of the hydrogels

LM pectin-based hydrogel films were developed via ionotropic gelation using CaCl_2_ (70 mg/g film) as the crosslinker and theophylline (30 mg/g film) as a model drug. A detailed methodology can be found in our previous studies [1,35,36]. Five different plasticizer solutions (CO, PVP, PEG400, PEG1000, and PEG1500) at 5% (w/w) were added to 2% (w/v) LM-pectin solutions, resulting in a pectin-to-plasticizer ratio of 2:1 in the final film composition. CO, as a hydrophobic triglyceride, was expected to generate microdomain-induced diffusion pathways; PVP, with water-affinitive lactam units, to promote moisture-driven plasticization; and PEG variants of different MWs (400/1000/1500), with hydroxyl-terminated chains, to establish hydrogen-bonding interactions with pectin. These distinct molecular features were anticipated to differentially affect crystallinity, hydration, and ultimately drug-release kinetics.

PVP and all PEG variants were magnetically stirred for 2 h, whereas CO, due to its higher viscosity and hydrophobicity, required 4 h of mixing. All mixing procedures were carried out at 150 rpm and ambient temperature. Following plasticizer incorporation, an aqueous solution of theophylline was added, and the mixtures were stirred under dark conditions for an additional 24 h at 150 rpm to ensure homogeneous drug distribution. The resulting formulations were cast onto 10 mL of 0.7% (w/w) CaCl_2_ solution and subsequently dried at 27 ± 1 °C under gentle agitation. The final hydrogel films were designated as P–x, where “P” denotes the LM-pectin matrix and “x” indicates the incorporated plasticizer.

### 2.3. Structural and thermal characterization

The FT-IR spectra of the hydrogels were obtained in the range of 4000–600 cm^−1^ using the KBr pellet or attenuated total reflectance (ATR) technique with the help of a PerkinElmer Spectrum One FT-IR Spectrometer (PerkinElmer Inc., Beaconsfield, United Kingdom) at room temperature.

The thermal properties of the films were analyzed using a DSC 4000 (PerkinElmer Instruments, USA) under a nitrogen atmosphere with a flow rate of 50 mL/min. The samples were sealed in aluminum pans and heated at a rate of 10 °C/min over a temperature range of −50 °C to 300 °C [1].

### 2.4. Determination of swelling behavior

Hydrogel films were cut into 15-mm-sized pieces using a template and placed in a vacuum oven at 30 °C overnight under vacuum to remove excess moisture. The dried samples were then immersed in a pH 6.4 tris buffer solution, simulating a biological environment, and weighed at predetermined time intervals until a constant weight was achieved [1,6,37]. All experiments were conducted under identical conditions and repeated at least three times for reproducibility. The swelling ratio was calculated using Equation 1.


Equation 1 $$\text{Swelling }(\%)=\frac{{\text{W}}_{\text{S}}-{\text{W}}_{\text{D}}}{{\text{W}}_{\text{D}}}\times 100,$$

where W_S_ represents the weight of the sample after hydration and W_D_ denotes the weight of the dried sample.

### 2.5. Determination of in vitro drug release profile and kinetics

Prior to the release experiments, hydrogel films were dried in a vacuum oven at 30 °C until an equilibrium (constant) weight was reached, following the same procedure described for the swelling studies (Section 2.4). Circular hydrogel disks, 7 mm in diameter, were placed in vials containing tris biological buffer solution at pH 6.4. To determine the in vitro drug release profiles, the vials were incubated on an orbital shaker set at 25 ± 1 °C and stirred at a constant speed of 100 rpm. At predetermined time intervals, 700-μL samples were withdrawn from the release medium and analyzed using a UV spectrophotometer at a wavelength of 271 nm. The measured absorbance values were used to calculate the percentage of drug released, utilizing a previously established calibration curve [1,38,39]. All experiments were conducted under identical conditions and repeated at least three times to ensure the reliability and reproducibility of the results. The release profiles of hydrogels were analyzed using five different kinetic models—zero-order, first-order, Korsmeyer–Peppas, Higuchi, and Hixson–Crowell [39,40].

### 2.6. Statistical analysis

Statistical analyses were performed using Minitab 16 software (Minitab Inc., State College, PA, USA). The swelling and drug release data were analyzed using two-way analysis of variance (ANOVA) to evaluate the effects of formulation variables. All ANOVA tests were followed by Tukey’s post-hoc test for pairwise and simultaneous comparisons of independent variables at a 95% confidence level (p < 0.05).

## 3 Results and discussion

**Figure 1:**
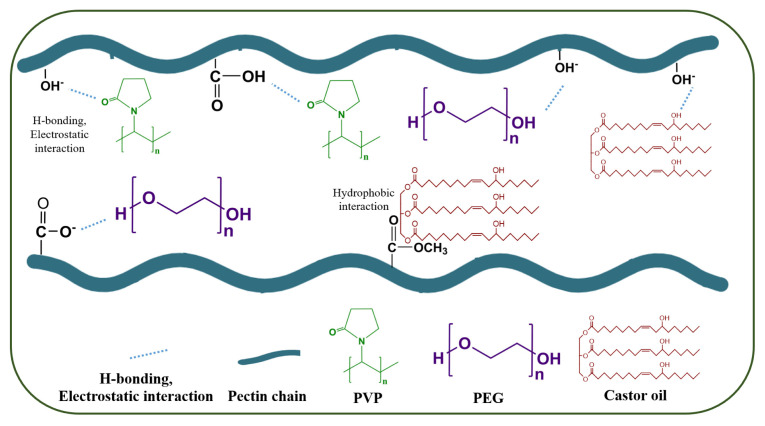
Schematic representation of the potential interactions between pectin and CO, PEG, and PVP.

In the present study, theophylline-loaded hydrogel films incorporating five distinct plasticizers (CO, PEG400, PEG1000, PEG1500, and PVP)—coded as P–CO, P–PEG400, P–PEG1000, P–PEG1500, and P–PVP, respectively—were developed to investigate the influence of plasticizer type on controlled drug release mechanisms. The schematic illustration in Figure 1 highlights the distinct interaction modes and possible interactions between the pectin matrix and each plasticizer. While PEG and PVP primarily interact via hydrogen bonding through hydroxyl and lactam groups, respectively, CO is suggested to participate through both ester-based hydrogen bonding and hydrophobic interactions. These molecular-level interactions form the structural foundation of the hydrogel network, influencing its swelling and drug release behavior. The proposed interaction mechanisms are further supported by the FTIR and DSC analyses presented in the subsequent sections. These films were synthesized with a cost-effective sol–gel process, wherein dynamic dual ionic bonds served as the primary cross-linking element [35,41]. The resultant hydrogel matrices were crack-free, homogeneous, and transparent, characteristics that may facilitate real-time optical monitoring in biomedical applications.

### 3.1. FTIR analysis

**Figure 2:**
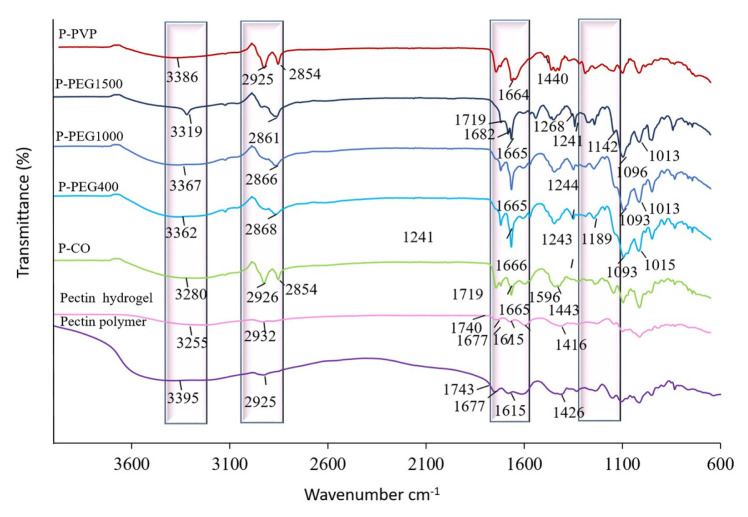
FTIR spectra of the hydrogels.

The FTIR spectra shown in Figure 2 demonstrated the nature of physicochemical interactions among LM-pectin, CaCl_2_, theophylline, and plasticizers in the hydrogel matrix. Neat pectin exhibited a broad O–H stretching band (3200–3600 cm^−1^), alongside characteristic peaks at 1743 cm^−1^ (C=O stretching of ester groups), 1677 cm^−1^ (COO^−^ asymmetric stretching), and 1426 cm^−1^ (C–H bending of acetyl and methoxy groups), consistent with previous reports [1]. Upon theophylline loading, additional bands near 1665 cm^−1^ (C=C and C=N stretching) emerged, with minor shifts indicative of weak theophylline–Ca^2+^ complexation [35,42,43].

As shown in Figure 2, all plasticized hydrogels exhibit broad O–H stretching peaks in the range of 3250–3400 cm^−1^, highlighting the abundance of OH^–^ groups. In the P-PEG hydrogels, shifts and intensity variations in the O–H region correspond to an increase in PEG MW (400–1500), suggesting enhanced hydrogen bonding and greater chain entanglement. The intensified C–H stretching vibrations (2800–2900 cm^−1^) indicate a higher methylene content as the MW increases [44,45]. Although characteristic C–H stretching vibrations around 2925 and 2854 cm^−1^ are observed for all PEG formulations, a clear correlation between MW and intensity is not distinctly evident, potentially due to scaling variations or overlapping contributions in the spectra. Additionally, peaks observed in the 1000–1200 cm^−1^ range, characteristic of C–O–C stretching, confirm the presence of ether linkages in PEG [44].

According to the literature, CO introduces distinct bands at 3280–3400 cm^−1^ (O–H stretching) and 2800–3006 cm^−1^ (C–H stretching of long aliphatic chains). Notable triglyceride peaks around 3280 and 1665 (indicating =C–H and C=C, respectively), as well as 2926 and 2854 cm^−1^ (C–H stretching of methylene groups), and 1740 and 1163 cm^−1^ (C=O and C–O stretching), confirm the triglyceride nature of the oil [46–50]. In the P–CO hydrogel, overlapping ester (1743 and 1719 cm^−1^) and shifted peaks at 1443 cm^−1^ (from the 1426 cm^−1^ peak of P) and 1665 cm^−1^ (from the 1653 cm^−1^ peak of CO) suggest reinforced hydrogen bonding [25,46–50]. The shift of the carboxylate band from 1615 to 1596 cm^−1^ confirms ionic crosslinking of Ca^2+^–pectin chains [1].

The FTIR spectrum of PVP exhibits broad N–H stretching (3300–3400 cm^−1^) and a distinct C=O peak (1644 cm^−1^) from the pyrrolidone ring [51]. The 1000–1300 cm^−1^ region corresponds to C–N and C–C stretching [52]. In neat pectin, the O–H peak appears at 3395 cm^−1^, whereas in PVP it appears at 3407 cm^−1^ [51]. Within the P–PVP hydrogel, these bands shift to lower wavenumbers (3386 cm^−1^ for O–H and 1664 cm^−1^ for C=O), underscoring enhanced hydrogen bonding [51].Furthermore, the splitting of C–H peaks into two bands (2925 and 2854 cm^−1^) and the shift of the pectin COO^_^ peak from 1426 to 1440 cm^−1^ confirm newly formed intermolecular interactions [51].

### 3.2. DSC analysis

To further investigate the effect of plasticizers on the internal structure of pectin hydrogel, formulations were subjected to differential scanning calorimetry (DSC) analysis (Table S1). The melting point (T_m_) and enthalpy of fusion (ΔH_m_) were evaluated to gain insight into polymer–plasticizer interactions and the degree of crystalline organization within the network.

Among the hydrogels analyzed, P–CO exhibited the lowest T_m_ (218°C), indicating that the bulky and hydrophobic triglyceride chains of CO disrupt the ordered arrangement of pectin chains and reduce thermal stability [53]. Although CO contains hydroxyl groups capable of hydrogen bonding with pectin, its overall effect is dominated by hydrophobic interactions, which promote chain mobility and contribute to a more flexible but thermally less stable matrix [54].

In contrast, hydrogels containing PEG400, PEG1000, PEG1500, and PVP showed higher T_m_ values. P–PEG400 exhibited a T_m_ of 223 °C, P–PEG1000 and P–PVP both displayed 226 °C, and P–PEG1500 reached 227 °C. These results reflect enhanced network stability and partial crystallinity. This thermal enhancement is primarily attributed to hydrogen bonding between PEG hydroxyl end groups and pectin carboxylate groups, which strengthens the network while allowing limited flexibility [55,56]. The shift in T_m_ from 223 °C to 227 °C with increasing PEG MW illustrates the ability of longer PEG chains to reinforce crystalline domains more effectively. PVP, due to its branched structure and abundant hydrogen-bonding sites, exhibited a similarly high T_m_ (226°C), underscoring its effectiveness as a structural stabilizer [56–58].

The enthalpy of fusion (ΔH_m_) values further support these observations (Table S1). P–PEG400 displayed the lowest ΔH_m_ (8.38 J/g), indicative of a disrupted crystalline framework due to its short chain length and strong plasticizing effect [6]. In contrast, P–PEG1000 (38.72 J/g), PEG1500 (45.10 J/g), and P–PVP (52.54 J/g) showed significantly higher ΔH_m_ values, indicating stronger intermolecular interactions and higher degrees of crystallinity. The increased ΔH_m_ for PEG1000 and PEG1500 reflects the formation of more ordered regions, while PVP’s hydrogen bonding capacity likely facilitates crystal lattice organization within the matrix. P–CO exhibited an intermediate ΔH_m_ value (36.96 J/g), representing a balance between the disordering influence of its lipidic chains and the stabilizing effect of its hydroxyl groups.

Taken together, the thermal properties of the hydrogels reflect the dual roles of plasticizers in modulating both flexibility and structural integrity. PEG400 acted as a strong plasticizer but reduced thermal resistance, whereas PEG1000, PEG1500, and PVP promoted crystalline stabilization while retaining some network flexibility. Among the plasticizers tested, CO offered a unique balance—combining moderate thermal stability with enhanced matrix flexibility—highlighting its versatility as a multifunctional modifier in LM-pectin-based hydrogel systems.

### 3.3. Swelling ratio studies

**Figure 3:**
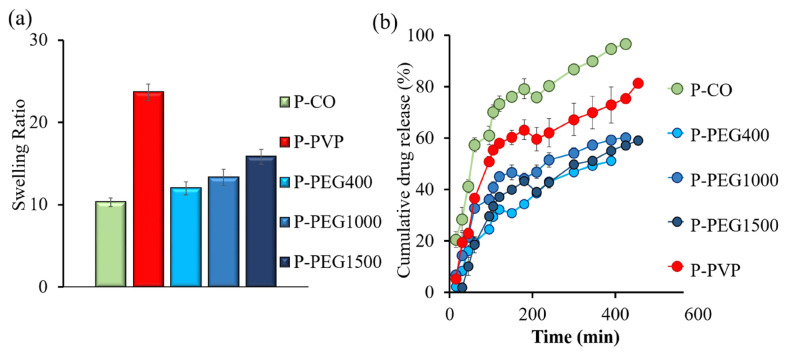
(a) Swelling ratio of the hydrogels, (b) Drug release profile of the hydrogels.

Figure 3a presents the equilibrium swelling ratios (SRs) of all LM-pectin-based hydrogel formulations. Among them, the P–PVP hydrogel exhibited the highest SR (23.7), which is attributed to the strong hygroscopic character of PVP [59]. The polar lactam and carboxyl groups in PVP promote extensive hydrogen bonding with water molecules. Additionally, the absorbed water may act as a secondary plasticizer, enhancing polymer chain mobility and improving matrix flexibility [51,60]. A similar effect was previously observed in our pectin–glycerol systems, where glycerol disrupted intermolecular hydrogen bonds, promoted chain mobility, and facilitated both water uptake and permeability [35,36].

In contrast, P–CO hydrogels showed the lowest SR (12.7), due to the pronounced hydrophobicity of CO, which inhibits water affinity and reduces solvent ingress. The dense aliphatic chains of CO restrict hydration, while the relatively high crystallinity observed in DSC analysis contributes further rigidity to the network. This combination of limited hydrophilicity and crystalline constraint explains the minimal swelling behavior observed in P–CO formulations.

Statistical analysis (one-way ANOVA, p = 0.005) confirmed that the plasticizer type had a significant influence on the SR. Post-hoc Tukey tests indicated that P–PVP exhibited significantly higher SR values than P–CO and PEG-based systems (p < 0.05), whereas no significant differences were detected among PEG400, PEG1000, and PEG1500 formulations (p > 0.05).

PEG-containing hydrogels exhibited intermediate SRs, which were modulated by the MW of the PEG used. PEG400, with its short and flexible chains, promotes rapid initial water uptake by increasing segmental motion. However, its smaller molecular size also renders it more susceptible to leaching from the matrix, thereby reducing the final swelling capacity. In contrast, PEG1000 and PEG1500 introduce more entangled structures that partly impede water penetration but allow sustained hydration due to PEG’s inherent hydrophilicity.

DSC analysis revealed that increasing PEG chain length led to elevated crystallinity and higher ΔH_m_ values. Nonetheless, longer PEG chains result in fewer polymer–PEG interactions per unit weight, effectively reducing crosslink density. This trade-off between enhanced crystalline ordering and diminished crosslinking explains the comparable SR values observed for P–PEG1000 and P–PEG1500, which occupy a balance between restricted swelling and improved water retention [44,61].

### 3.4. Drug release pattern and release kinetics of the hydrogels

When plasticizers are incorporated into polymeric matrices, they influence chain conformation and introduce capillary pathways, effectively modulating hydrophilicity, water diffusivity, and drug transport. This structural modulation leads to increased solubility and higher diffusion coefficients within the hydrogel network [7,62,63]. Among the formulations tested, P–CO exhibited the highest cumulative drug release, despite its relatively low SR (Figure 3b). This behavior can be attributed to its semicrystalline structure and the presence of hydrophobic microdomains, which generate voids that enable sustained diffusion. DSC and FTIR analyses confirm increased matrix rigidity coexisting with permeable regions, resulting from hydrophobic interactions between CO’s ester chains and pectin.

One-way ANOVA followed by Tukey’s post-hoc tests showed that plasticizer type had a statistically significant effect on cumulative release (p < 0.05). P–CO and P–PVP represented the fastest and the most sustained profiles, respectively. Within the PEG series the ordering was PEG400 > PEG1000 ≈ PEG1500 (Tukey: PEG400 vs. PEG1000/PEG1500, p < 0.05; PEG1000 vs. PEG1500, p > 0.05).

Across all plasticizer-containing formulations, the release was not governed by simple concentration-dependent (first-order) kinetics, as the first-order plots showed poor linearity (R^2^ = 0.46–0.61), indicating that mechanisms beyond concentration-driven release dominate (Figure S1).

The release profile corresponded well with the Korsmeyer–Peppas (R^2^ = 0.9164, n = 0.4373) and Hixson–Crowell (R^2^ = 0.9302) models, indicating a dual mechanism governed by diffusion and surface-mediated erosion (Figures S2 and S3; Table S2). The n-value of 0.4373 in the Korsmeyer–Peppas model indicates primarily diffusion-driven release, while the Hixson–Crowell model reflects additional contributions from changes in surface area and particle diameter over time. Despite its low SR, the total release was elevated and sustained over an extended period, consistent with the ability of CO to fill microvoids, diminish water retention, and facilitate controlled diffusion [64]. The presence of hydrophobic domains appears to induce water molecules to form more ordered hydrogen-bond networks, reducing the internal energy of the system and prolonging the release timeframe [65,66]. The lipophilic nature of CO further slowed diffusion, contributing to prolonged release.

To clarify the comparative behavior among PEG-based systems, we relate the release to structure evidenced in Sections 3.1 and 3.2 (Figure 1; Table S1). PEG400 shows reduced crystallinity and enhanced hydration—reflected by lower ΔH_m_/broadened endotherm in DSC and a broadened O–H band in FTIR—thereby increasing chain mobility and promoting a faster, non-Fickian transport (diffusion + polymer relaxation). PEG1000 displays an intermediate pattern in which moderate crystallinity and balanced hydration support non-Fickian diffusion governed by both polymer relaxation and diffusional transport. PEG1500 forms a denser, more ordered network that limits chain mobility and favors diffusion-dominated transport. This structure–property rationale is fully consistent with the findings given above (PEG400 > PEG1000 ≈ PEG1500).

In the case of P–PEG400, a strong correlation with the Higuchi model (R^2^ = 0.9759) and adequate fit with the zero-order and Hixson–Crowell models were observed. Its high release exponent (n = 0.8268) in the Korsmeyer–Peppas model points to a non-Fickian mechanism involving both diffusion and polymer relaxation and erosion [67]. Supporting data from swelling tests show moderate hydration [68], while DSC results reveal a partially ordered structure that permits controlled drug mobility. Similar observations were reported by Mundada and Avari [69], where glycerol-plasticized gum copal films showed higher release rates compared to those plasticized with PEG400 (Figures S2–S4; Table S2).

P–PEG1000 also exhibited non-Fickian behavior (n = 0.61), with strong model fits (Higuchi R^2^ = 0.8933 and Korsmeyer–Peppas R^2^ = 0.8644) (Figures S2 and S5; Table S2) [70,71]. Its moderate crystallinity, as observed in DSC analysis, combined with increased chain entanglement and balanced hydration, enables effective diffusion consistent with previous studies on pectin-based networks [70,72–74]. In line with ANOVA/Tukey, the cumulative release of P–PEG1000 was statistically indistinguishable from P–PEG1500 (p > 0.05), placing PEG1000 between PEG400 and PEG1500 in terms of performance.

Compared to other PEG-based systems, P–PEG1500 followed a diffusion-dominant release, best fitting the Higuchi model (R^2^ = 0.9243) forming denser, less mobile films with lower swelling capacity. Although it demonstrated low swelling, FTIR analysis revealed the presence of sufficient hydrophilic regions within the dense matrix to enable drug transport—potentially through pore formation or localized hydration channels [75].

The present study demonstrated the pivotal role of plasticizer selection in modulating the physicochemical properties, structural organization, and drug release behavior of LM-pectin-based hydrogels. Each plasticizer imparted distinct effects on the network architecture and release kinetics. Among the systems tested, P–CO hydrogels exhibited the highest cumulative drug release, a result of reduced polymer–polymer cohesion and disrupted pectin packing, which promoted matrix flexibility and sustained diffusion.

In contrast, PVP-based hydrogels presented slower and more prolonged release profiles, attributed to enhanced crystallinity and increased network rigidity. PEG-containing hydrogels showed molecular-weight-dependent behaviors: PEG400 allowed for increased mobility owing to its shorter chains, reduced crystallinity, and improved hydration capacity, thus promoting accelerated release through non-Fickian transport; PEG1000 displayed an intermediate profile, where moderate crystallinity and balanced hydration enabled non-Fickian diffusion influenced by both polymer relaxation and diffusional transport; conversely, PEG1500 further impeded release due to substantial network densification and decreased chain mobility, despite the existence of crystalline domains. While DSC data supported the crystalline characteristics of higher-MW PEG formulations, the reduced permeability observed in P–PEG1500 reflected a tightly packed structure with restricted diffusional pathways.

These findings underscore the intricate interplay between plasticizer chemistry, network structure, and water–polymer interaction in governing drug release. Future investigations should focus on the long-term stability of these systems and explore strategies such as dual-plasticizer combinations or the incorporation of functionalized additives to fine-tune both release kinetics and mechanical performance. Given their inherent biocompatibility, controllable swelling, and adaptable release characteristics, LM-pectin hydrogels offer substantial translational potential for topical biomedical applications, including dermal patches, wound dressings, and mucosal drug delivery platforms.

## 4 Conclusion

Our study underscores the pivotal role of plasticizer type and MW in modulating the structural integrity, physicochemical behavior, and drug release performance of LM-pectin-based hydrogel systems. The incorporation of distinct CO, PVP, and PEG (MW 400–1500) resulted in markedly different network architectures and release kinetics. Among the systems tested, P–CO hydrogels achieved the highest cumulative drug release, primarily due to the disruption of pectin chain packing by hydrophobic triglyceride domains, which enhanced matrix flexibility and diffusion. Conversely, PVP-based hydrogels exhibited prolonged and controlled release profiles, attributable to their increased crystallinity and network stiffness. PEG-modified systems revealed MW-dependent release dynamics: PEG400 yielded limited release due to reduced swelling, whereas PEG1500 restricted diffusion via intensified secondary interactions and enhanced crystalline domains, as substantiated by DSC thermograms. In contrast, PEG1000 exhibited an intermediate release pattern, showing cumulative values positioned between PEG400 and PEG1500. This behavior stems from its moderate crystallinity and balanced hydration, which together maintain adequate chain mobility and enable a mixed diffusion–relaxation transport regime. Such a balanced structure facilitates sustained but not hindered release, consistent with the DSC and kinetic findings.

These observations suggest that plasticizer-mediated modulation of SR, network density, and crystallinity critically governs drug transport in pectin hydrogels. These insights contribute to the rational design of hydrogel matrices with tunable release characteristics. Future work should prioritize long-term stability assessment, exploration of synergistic plasticizer combinations, and surface-functionalization strategies to advance the translational potential of LM-pectin hydrogels in controlled drug delivery and biomedical interfaces.

## Supplementary information

### 1 Materials and methods

#### 1.1. Calculation of in vitro drug release kinetics

Drug release kinetics were analyzed using zero-order, first-order, Korsmeyer–Peppas, Higuchi, and Hixson–Crowell models, providing a comprehensive framework for understanding the mechanisms governing drug release from various formulations (Table S2). Each model was assessed graphically, and linear regression (based on the coefficient of determination, R^2^) was used to identify the best-fitting model for each hydrogel formulation.

The zero-order kinetic model, shown in Equation 1, assumes a constant release rate and is expressed as


Equation 1 $$Q_{\text{t}}=Q_{0}+Kot,$$

where *Q*_0_ is the concentration of drug released at time *t*, *Q*_0_ is the initial drug concentration in solution, and *K*_0_ is the zero-order release constant (unit: %/min).

The first-order model, represented in Equation 2, describes a release rate proportional to the amount of drug remaining and is given as


Equation 2 $$log_{C}=log_{C0}-K1_{t}/2.303,$$

where *C* denotes the percentage of drug remaining at time *t*, *C*0 is the initial drug concentration, and, *K1* is the first-order release constant (1/min).

The Korsmeyer–Peppas model (Equation 3) is used to describe both diffusion-controlled and anomalous drug release mechanisms:


Equation 3 $$\frac{M_{t}}{M_{\infty }}=K_{P}t^{n},$$

where $\frac{M_{t}}{M_{\infty }}$ refers to the fraction of drug released at time *t*, *K_P_* is the Korsmeyer release constant, and *n* is the diffusion exponent indicating the mechanism:

n ≤ 0.45: Fickian diffusion
0.45 < n < 0.89: Non-Fickian (anomalous)
n = 0.89: Case-II transport
n > 0.89: Super case-II transport

The Higuchi model, which describes release based on diffusion through a porous matrix, is expressed as


Equation 4 $$Q=K_{H}t^{1/2},$$

where *Q* is the cumulative amount of drug released at time *t* and *K_H_* is the Higuchi dissolution constant (%/√min). This model assumes drug release is governed by Fickian diffusion.

Finally, the Hixson–Crowell model, which accounts for changes in surface area and particle size as the drug dissolves, is represented by


Equation 5 $${W_{0}}^{\frac{1}{3}}-{W_{t}}^{\frac{1}{3}}=K_{HC}t,$$

where *W*_0_ represents the initial amount of drug, *W*_1_ is the remaining amount of drug at time *t*, and *K_HC_* denotes the Hixson release constant.

The data for each model were graphically represented to provide a systematic approach to determining the most suitable model for describing the drug release behavior of the hydrogels.

### 2 Results and discussion

#### 2.1. Calculation of in vitro drug release kinetics

**Table S1:** DSC data for drug theophylline, P–CO, P–PVP, P–PEG400, and P–PEG1000.

Code	P–CO	P–PVP	P–PEG400	P–PEG1000	P–PEG1500
**Melting temperature (T _m_) (**° **C)**	218	226	223	226	227
**Melting enthalpy (ΔH _m_) (J/g)**	36.96	52.54	8.38	38.72	45.10

**Table S2:** The regression coefficient of the kinetic models fitted for P–CO, P–PVP, P–PEG400, P–PEG1000, and P–PEG1500. The bold R^2^ values indicate the highest coefficient of determination for each formulation, representing the best-fitting kinetic model among the tested models.

Code	Zero order (ZO)		First order (FO)		Korsmeyer–Peppas (KP)		Higuchi (H)		Hixon–Crowell (HC)		Best fit
	Ko	R ^2^	K1	R ^2^	n	R ^2^	K_ _H_	R ^2^	K_ _HC_	R ^2^	
**P–CO**	0.157	0.7701	0.0011	0.6074	0.4373	**0.9164**	4.2971	0.8933	0.0059	**0.9302**	KP, HC
**P–PVP**	0.156	0.7381	0.0015	0.4854	0.6357	0.8344	4.4706	**0.8666**	0.0033	0.839	H
**P–PEG400**	0.237	**0.9005**	0.0023	0.5874	0.8268	**0.9011**	5.986	**0.9759**	0.0024	**0.9355**	ZO, KP, HC, H
**P–PEG1000**	0.180	0.7761	0.0015	0.525	0.6139	**0.8644**	4.922	0.8933	0.0024	0.8393	KP
**P–PEG1500**	0.192	0.8014	0.002	0.4623	0.8079	0.8084	5.5514	**0.9243**	0.0024	0.8758	H

Figure S1 The theoetical lines fitted to the experimental points according to the zero-order kinetic model obtained from the analysis of the theophylline release from the hydrogel.

Figure S2 The theoretical lines fitted to the experimental points according to the first-order kinetic model obtained from the analysis of the theophylline release from the hydrogel.

Figure S3 The theoretical lines fitted to the experimental points according to the Korsmeyer–Peppas kinetic model obtained from the analysis of the theophylline release from the hydrogel.

Figure S4 The theoretical lines fitted to the experimental points according to the Higuchi kinetic model obtained from the analysis of the theophylline release from the hydrogel.

Figure S5 The theoretical lines fitted to the experimental points according to the Hixon–Crowell kinetic model obtained from the analysis of the theophylline release from the hydrogel.
