# Supplementary figures and images for: Influence of plasticizer type on the structure and drug release characteristics of LM-pectin hydrogels
Source: Turk J Chem. 2025 Nov 25;50(1):1–11. doi: 10.55730/1300-0527.3775 (PMC12965789; doi:10.55730/1300-0527.3775)

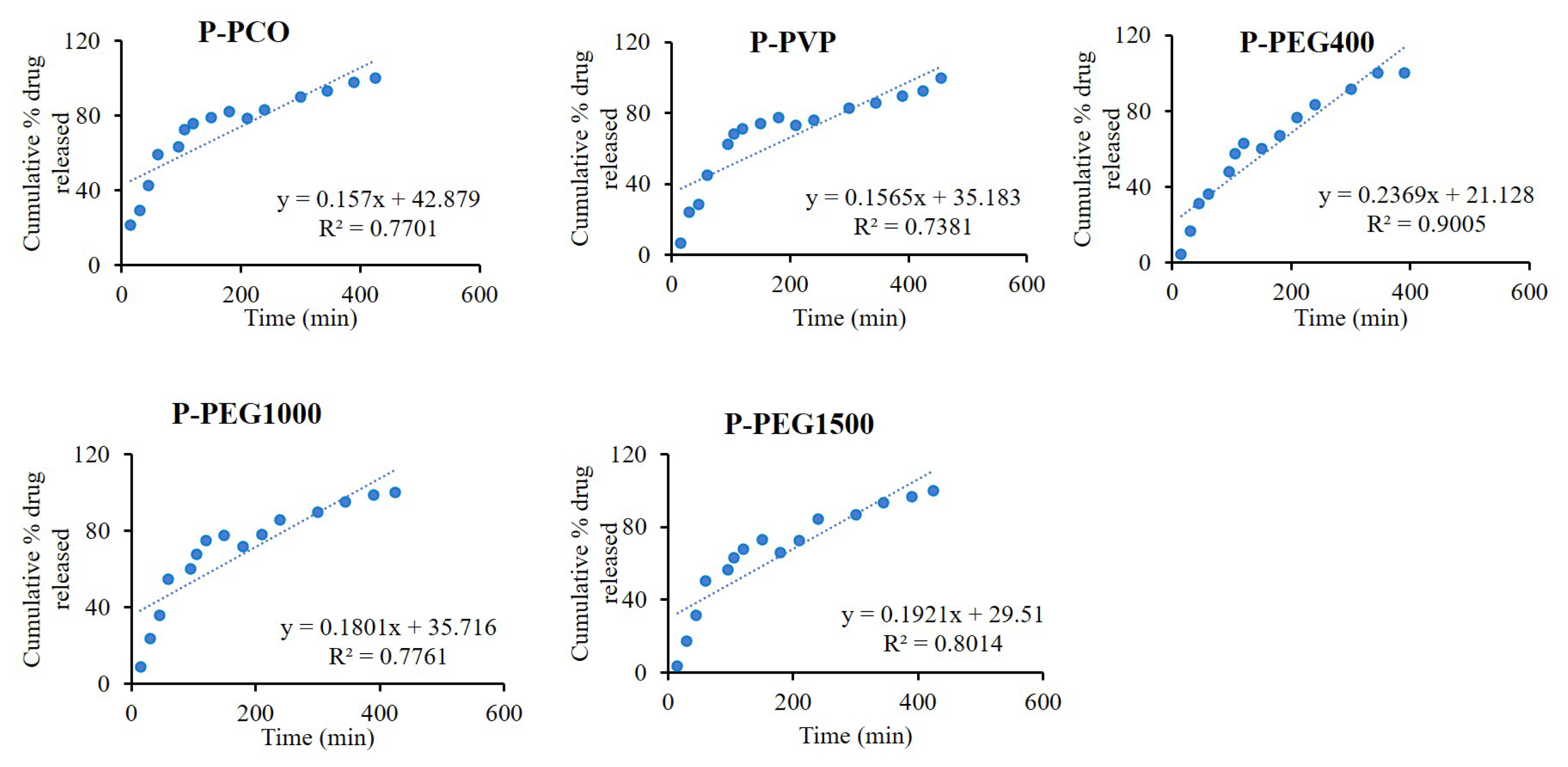

Supplement: Figure S1 — The theoetical lines fitted to the experimental points according to the zero-order kinetic model obtained from the analysis of the theophylline release from the hydrogel. [file tjc-50-01-1s1.tif]

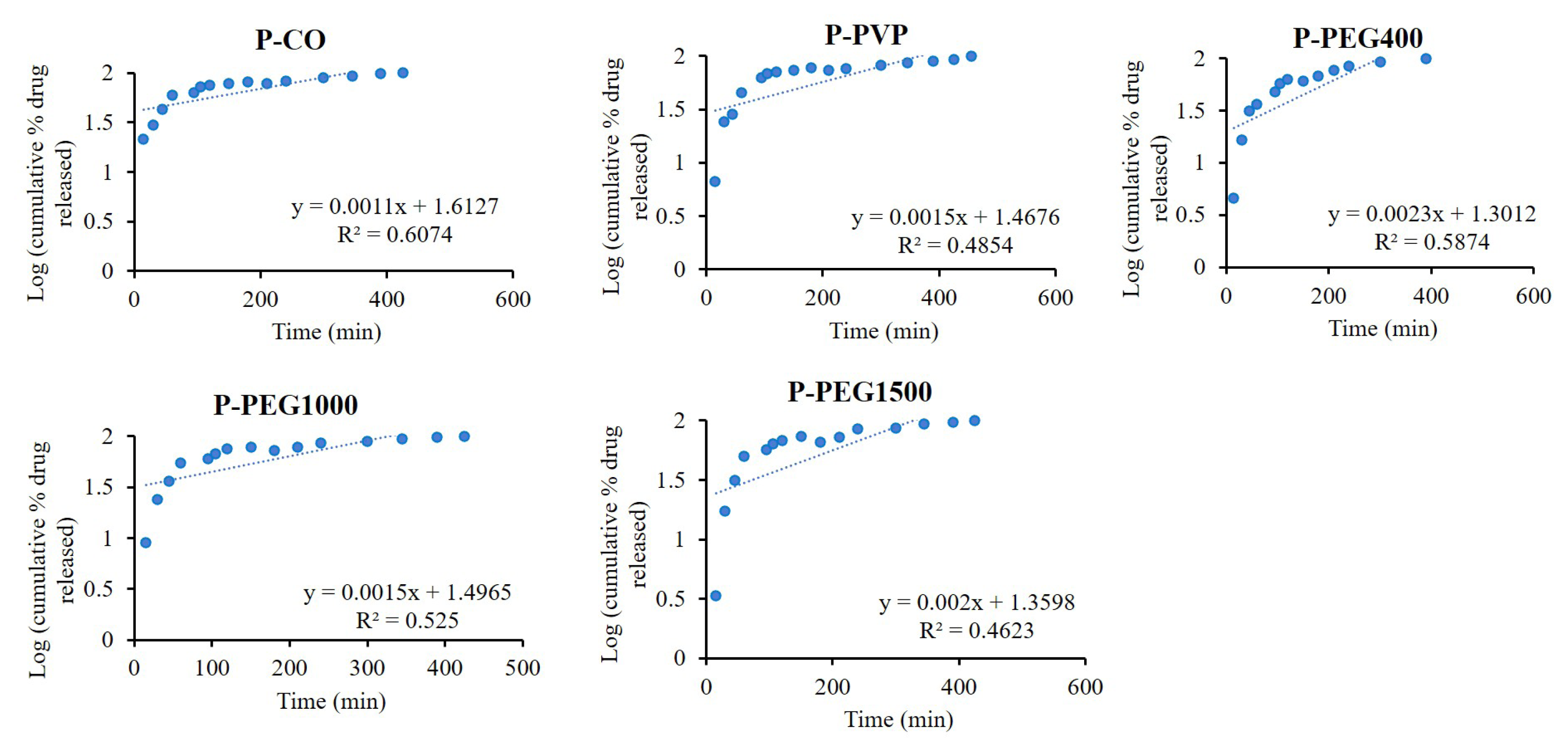

Supplement: Figure S2 — The theoretical lines fitted to the experimental points according to the first-order kinetic model obtained from the analysis of the theophylline release from the hydrogel. [file tjc-50-01-1s2.tif]

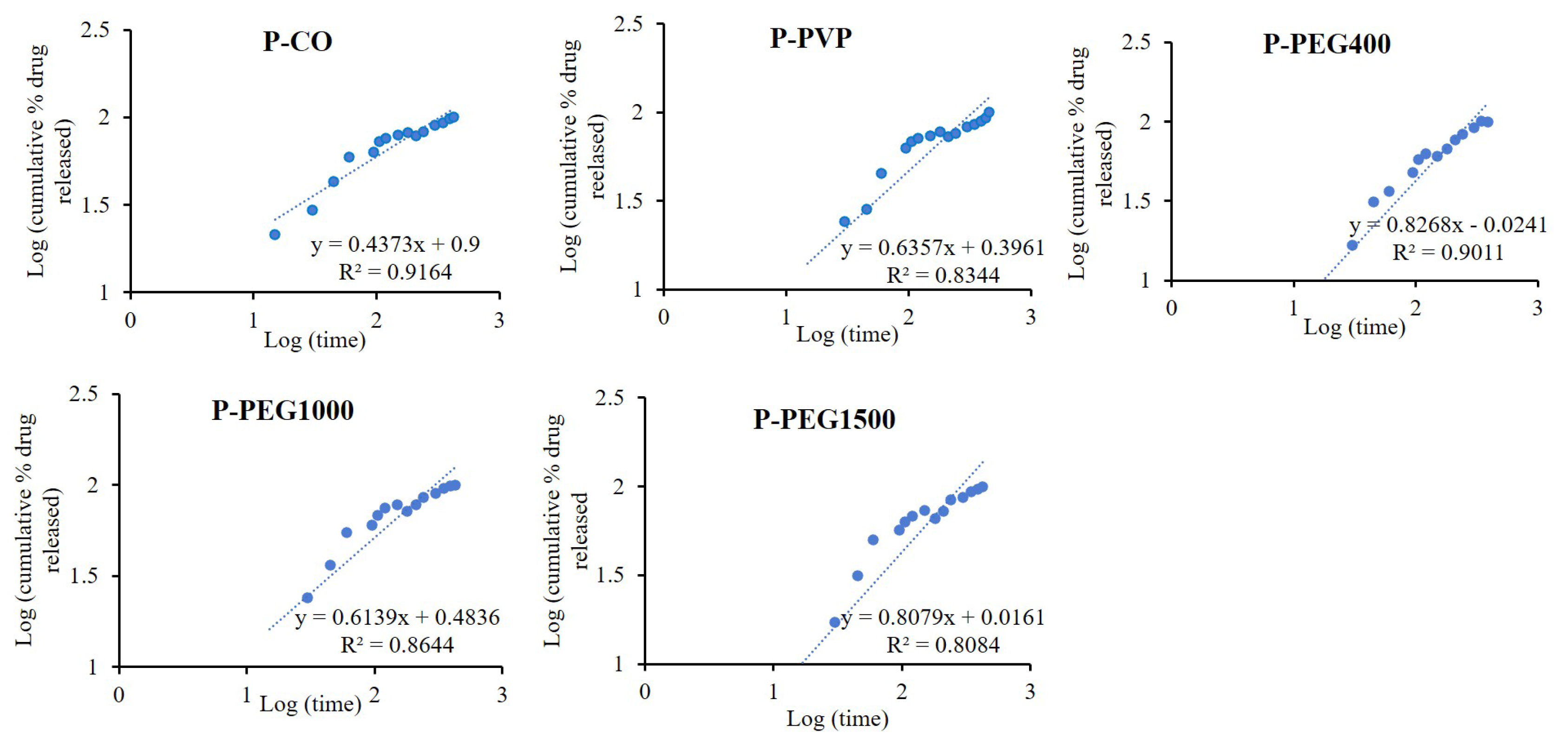

Supplement: Figure S3 — The theoretical lines fitted to the experimental points according to the Korsmeyer–Peppas kinetic model obtained from the analysis of the theophylline release from the hydrogel. [file tjc-50-01-1s3.tif]

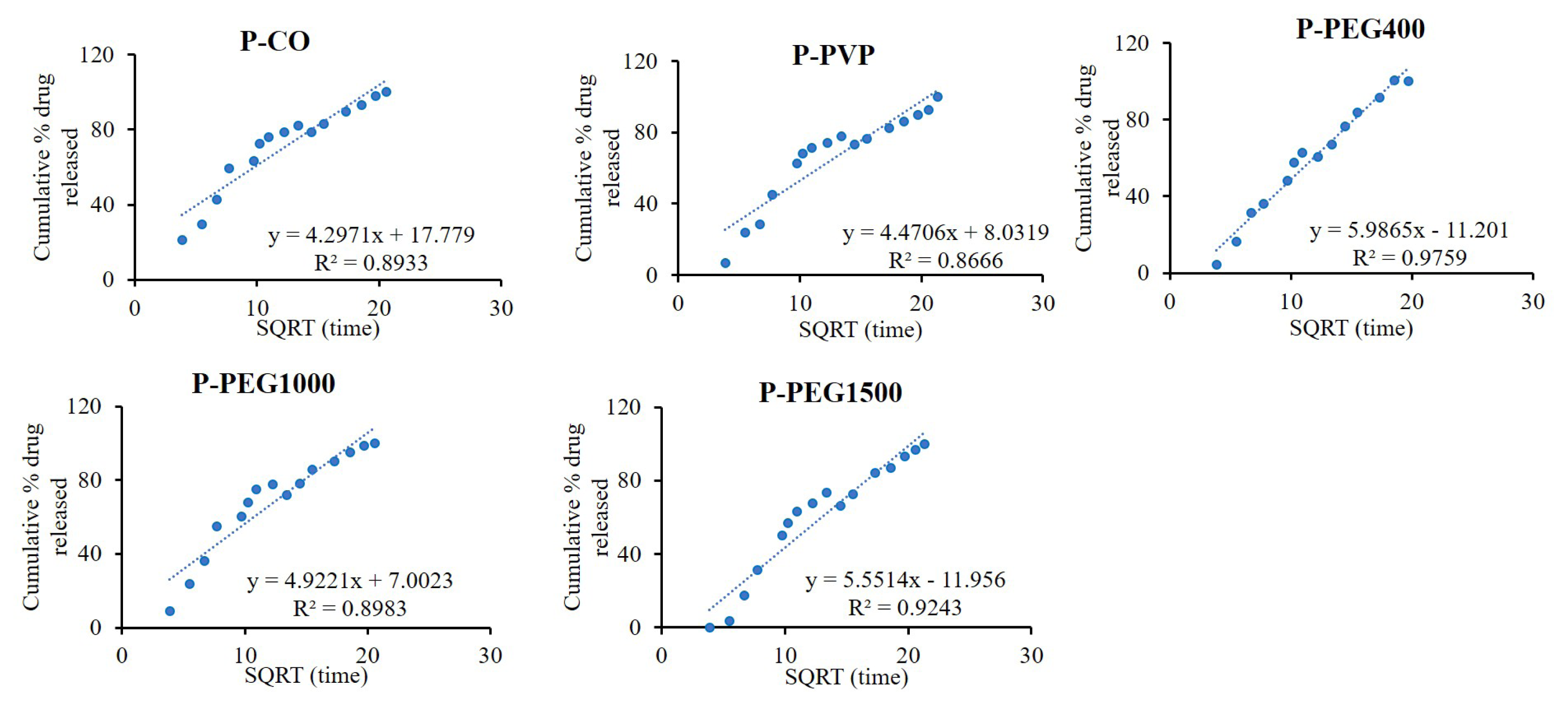

Supplement: Figure S4 — The theoretical lines fitted to the experimental points according to the Higuchi kinetic model obtained from the analysis of the theophylline release from the hydrogel. [file tjc-50-01-1s4.tif]

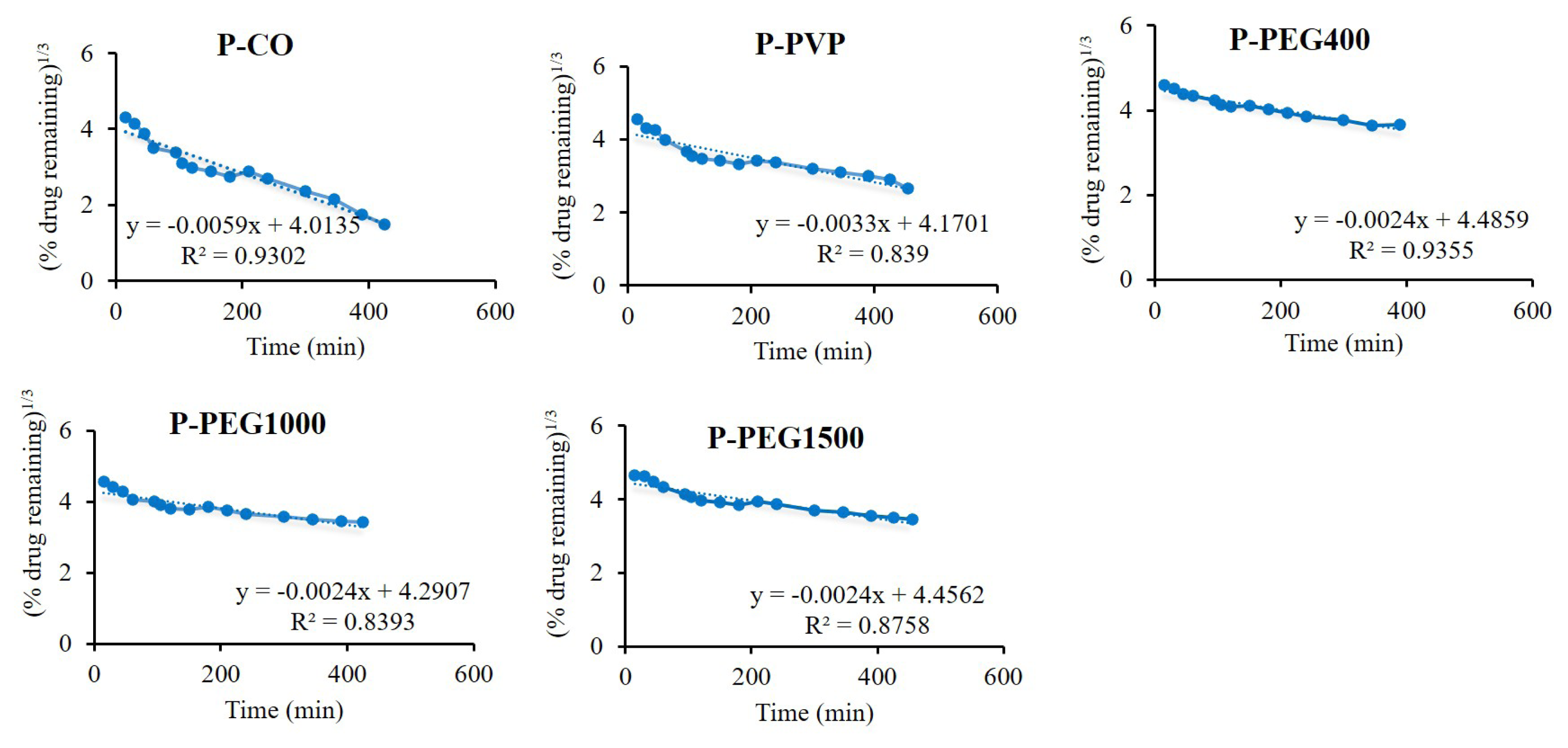

Supplement: Figure S5 — The theoretical lines fitted to the experimental points according to the Hixon–Crowell kinetic model obtained from the analysis of the theophylline release from the hydrogel. [file tjc-50-01-1s5.tif]
